# Efficient attention mechanisms for large language models

**DOI:** 10.1016/j.patter.2026.101594

**Published:** 2026-07-03

**Authors:** Yutao Sun, Zhenyu Li, Yike Zhang, Tengyu Pan, Bowen Dong, Yuyi Guo, Jianyong Wang

**Affiliations:** 1 Department of Computer Science, Tsinghua University, Beijing 100084, China

**Keywords:** language model, linear attention, sparse attention, hybrid model

## Abstract

Transformer-based architectures have become the prevailing backbone of large language models. However, the time and memory complexity of self-attention remains a fundamental obstacle to efficient long-context modeling. To address this limitation, recent research has introduced two principal categories of efficient attention mechanisms. Linear attention methods achieve linear complexity through kernel approximations, recurrent formulations, or fast-weight dynamics, thereby enabling scalable inference with reduced computational overhead. Sparse attention techniques, in contrast, limit attention computation to selected subsets of tokens based on fixed or dynamic strategies, enhancing efficiency while preserving contextual coverage. This survey provides a comprehensive overview of these developments, integrating both algorithmic innovations and hardware-level considerations. In addition, we analyze the incorporation of efficient attention into large-scale pre-trained language models, including both architectures built entirely on efficient attention and hybrid designs that combine local and global components. By aligning theoretical foundations with practical deployment strategies, this work aims to serve as a foundational reference for advancing the design of scalable and efficient language models.

## The bigger picture

The rapid evolution of large language models (LLMs) has fundamentally transformed how we interact with technology, enabling breakthroughs in coding, creative writing, and complex problem-solving. However, these models possess a significant efficiency bottleneck. As the length of the input text increases, such as when analyzing an entire book or a massive codebase, the computational resources required grow quadratically. In standard transformer models that use full attention, doubling the text length can require roughly four times the computation and memory, making long-context interactions prohibitively expensive and environmentally taxing. To sustain the growth of AI, we must move beyond these traditional, resource-heavy architectures toward more streamlined and scalable solutions.

This survey explores the frontier of “efficient attention” mechanisms, which aim to break this quadratic barrier. We categorize and explain emerging techniques such as linear attention, which simplify complex calculations to save time, and sparse attention, which focuses only on the most relevant information. Crucially, these innovations are not merely theoretical exercises; many of the methods we discuss have already been integrated into high-profile commercial models, proving their viability in real-world production. By bridging the gap between algorithmic theory, hardware deployment, and commercial implementation, this work provides a roadmap for the next generation of AI. Our analysis offers a foundational reference for researchers and engineers aiming to build scalable models that are not only more powerful but also more accessible and sustainable for the future.

## Teaser

This survey reviews efficient attention mechanisms for scalable long-context language modeling, with a focus on linear and sparse attention. It summarizes their algorithmic foundations, hardware considerations, and integration into large-scale pre-trained language models, providing a systematic reference for designing efficient transformer-based architectures.

## Introduction

Transformer-based architectures^1^ have become the *de facto* choice as the backbone of modern large language models (LLMs). Despite their success, the standard self-attention mechanism remains a significant computational bottleneck, with quadratic time and memory complexity with respect to input sequence length. This limitation poses substantial challenges for scaling LLMs to handle increasingly long contexts with both strong performance and high efficiency.

To address this, two major directions have emerged to reduce the time and space complexity of softmax attention. The first mechanism is linear attention,^2^^,^^3^^,^^4^^,^^5^^,^^6^^,^^7^ which seeks to reduce attention complexity by reparameterizing or approximating the softmax attention as linear operations. The second candidate is sparse attention,^8^^,^^9^^,^^10^^,^^11^^,^^12^ which restricts attention computation to a subset of the full key space based on fixed or dynamic sparsity patterns. While both approaches aim to improve efficiency, they differ significantly in formulation, design choices, and hardware implications. In parallel, GQA-style KV-cache compression methods^13^^,^^14^^,^^15^ are widely adopted in practice. Yet they do not reduce core attention computation and mainly provide fixed-ratio KV storage savings, so their overall impact is limited.

**Figure 1:**
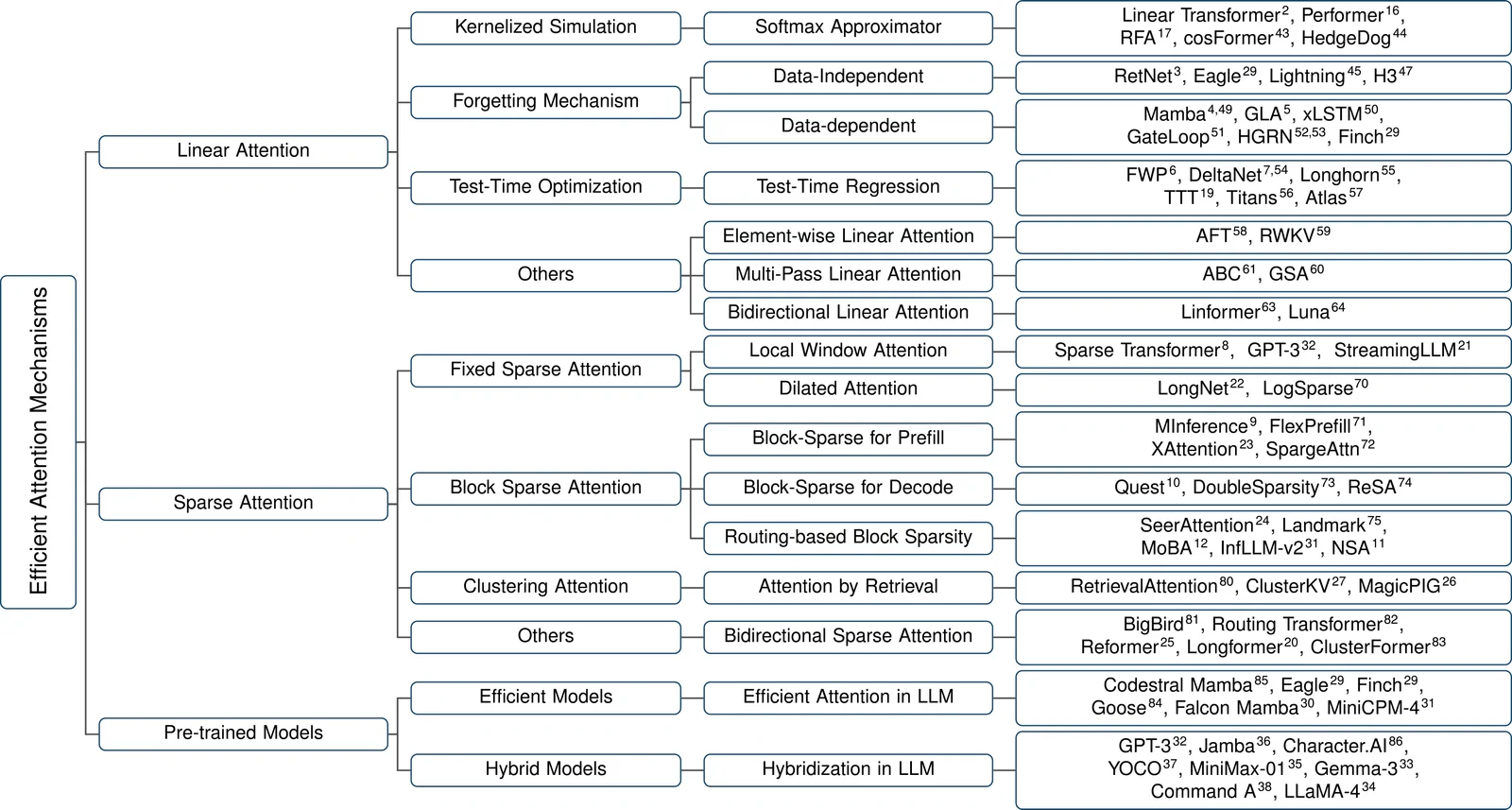
Taxonomy of efficient attention mechanisms

This survey provides a comprehensive review of recent developments in efficient attention mechanisms, with a dual focus on algorithmic principles and system-level implementation. Based on that, we also study the pre-trained LLM employing these efficient attentions. Figure 1 summarizes the taxonomy of efficient attention mechanisms based on representative prior work.

We categorize linear attention methods into three major paradigms. First, kernelized linear attention approximates the softmax kernel with inner products in a feature space, achieving linear complexity via random feature maps^16^^,^^17^ or fixed positive mappings.^2^ Second, recurrent linear attention with forgetting mechanisms introduces position-aware recurrence, enabling long-sequence modeling through data-independent^3^ or data-dependent decay,^4^^,^^5^ which control how past information fades over time. Third, fast-weight and meta-learning-based formulations reinterpret linear attention as a memory update process optimized online, where models such as DeltaNet^6^^,^^7^ and TTT^18^^,^^19^ incorporate fast-learning dynamics directly into the state evolution. We also examine hardware-friendly representations of linear attention—including parallel, recurrent, and chunkwise forms—highlighting their respective trade-offs in computational complexity, memory footprint, and compatibility with training or inference workflows.

We classify sparse attention into fixed-pattern sparsity, block sparsity, and clustering-based sparsity. Fixed-pattern sparsity adopts static token-level masks such as sliding windows, dilated positions, or designated global tokens, offering simplicity and hardware-friendliness.^8^^,^^20^^,^^21^^,^^22^ Block sparsity selects or routes attention at block granularity, either via heuristic scoring,^10^^,^^12^^,^^23^ trainable gating,^11^^,^^24^ enabling structured memory access and efficient GPU utilization. Clustering-based sparsity organizes key-value pairs using content-based or position-aware grouping methods, such as *k*-means or locality sensitive hashing (LSH), facilitating semantically aware retrieval with reduced memory overhead.^25^^,^^26^^,^^27^ These approaches differ in sparsity granularity, selection mechanism, and their alignment with hardware primitives like FlashAttention,^28^ and collectively represent the foundation for efficient long-context modeling in modern transformers.

There are recent efforts to integrate efficient attention mechanisms into industry-level pretrained language models. These include both pure efficient architectures, such as linear attention and state-space models, and hybrid designs that combine local and global attention patterns. Models like Eagle,^29^ Falcon Mamba,^30^ and MiniCPM4^31^ demonstrate the scalability of purely linear or sparse approaches to the multi-billion parameter scale, offering strong performance with constant-time inference. Meanwhile, hybrid models^32^^,^^33^^,^^34^^,^^35^^,^^36^^,^^37^^,^^38^ interleave dense, sparse, and local attention to balance computational efficiency with context modeling capacity, reflecting a growing trend toward compositional, hardware-aware attention designs in modern LLMs.

Our goal is to provide a unified framework for understanding the evolution of attention mechanisms under both algorithmic and hardware constraints, and how these designs are integrated into scalable LLM architectures. By connecting theoretical insights with practical implementations, we hope this survey offers a valuable reference for researchers and practitioners working toward efficient and deployable model design. Early surveys^39^ provided a broad overview of efficient transformer variants. More recent works^40^^,^^41^ further expanded the landscape by covering efficient long-context modeling strategies and attention variants. Compared with these works, our survey aims to provide a clearer organization of the design space, together with a more systematic analysis between modeling capability and hardware efficiency, and a set of practical takeaways for choosing among sparse, linear, and hybrid designs in long-context deployment.

This survey is motivated by a pivotal transition in the landscape of LLMs. While previous development was predominantly driven by a parameter-scaling paradigm aimed at maximizing capability, the current frontier has shifted toward an efficiency-centric orientation. As demands for million-token contexts and low inference cost, the quadratic time-space complexity of canonical self-attention constitutes the principal bottleneck inhibiting scalability. Consequently, the redesign of attention mechanisms has emerged as the critical frontier for architectural innovation. Synthesizing these developments is essential to bridge the disconnect between theoretical algorithmic design and the practical latencies imposed by modern hardware accelerators, thereby laying the groundwork for the next generation of scalable and commercially viable models.

## Linear attention

### Kernelized linear attention

Traditional linear attention methods seek to approximate the softmax-based attention mechanism in a way that scales linearly with sequence length. The core idea is to replace the expensive softmax computation with a kernel-based approximation of the attention weights. In standard self-attention, each output is a weighted sum of values *V* with weights given by a softmax over query-key similarities:

(Equation 1) $$\text{Attn}\left(Q,K,V\right)=\text{softmax}\left(QK^{⊤}\right)V\text{,}$$

where $Q,K,V\in R^{L\times d}$ (with *L* the sequence length and *d* the model dimension per head). The softmax yields weights $\propto \exp\;\left(q_{i}^{⊤}k_{j}\right)$ for query *q*_*i*_ and key *k*_*j*_. Kernelized linear attention instead finds a feature mapping *ϕ*(·) such that the softmax kernel is approximated by a simple dot-product in an induced feature space: *exp*(*q*^⊤^*k*)≈*ϕ*(*q*)^⊤^*ϕ*(*k*).^42^ Given such a *ϕ*, one can rewrite attention as

(Equation 2) $$O=\frac{\phi \left(Q\right)\left(\phi {\left(K\right)}^{⊤}V\right)}{\phi \left(Q\right)\left(\phi {\left(K\right)}^{⊤}1\right)}\text{,}$$

*ϕ*(·) is usually chosen to produce non-negative outputs since *exp*(·)’s value region is non-negative, a normalization divisor is also applied to mimic the softmax probabilities. This reformulation reduces complexity from *O*(*L*^2^*d*) to *O*(*Ld*^2^) (or even *O*(*Ld*) with suitable feature dimension reduction), since the expensive *L*×*L* attention matrix is never formed explicitly.

(1) Linear transformer^2^ replaces the softmax kernel with a fixed positive feature map. In practice, they set *ϕ*(*x*) = ELU(*x*)+1. ELU(·) is differentiable in the whole defined region, showing a better performance than naive ReLU(·) function.
(2) Performer^16^ introduces FAVOR+, a random features scheme that unbiasedly approximates the softmax kernel. It samples randomized feature maps *ϕ* so that *E*[*ϕ*(*Q*)*ϕ*(*K*)^⊤^] = *exp*(*QK*^⊤^). This yields a provably unbiased estimator of full softmax attention using only O(N) operations. In particular, performers use positive orthogonal random features, which reduce variance in the approximation.
(3) Random feature attention^17^ is a linear attention built via random Fourier features for the softmax kernel. Similar to performer, RFA leverages random mapping and triangular activation to approximate softmax. RFA further normalizes the queries and keys before random projection to reduce variance. RFA also has a variant, RFA-gate, which adds an optional gating mechanism for recency bias.
(4) cosFormer^43^ proposes to use cosine function to approximate softmax. Since *cos*(*a*+*b*) = *cosacosb*-*sinasinb*, cosFormer decomposes the cosine re-weighted attention $S_{ij}=Q_{i}^{\prime }K_{j}^{\prime }\;\cos\left(\frac{\pi }{2}\times \frac{i-j}{M}\right)$ into a linear attention form. *S*_*ij*_ represents the attention score between the *i*-th query and *j*-th key. $Q_{i}^{\prime }$ and $K_{j}^{\prime }$ are non-negative projections.
(5) HedgeDog^44^ leverages a spiky kernel *ϕ*(*x*) = *exp*(*Wx*+*b*) since it observes that the performance gap between the transformer and linear transformer is due to the lack of spiky and monotonic properties. HedgeDog shows better attention entropy and monotonicity.

### Linear attention with forgetting mechanism

A more recent line of work interprets attention through the lens of recurrent neural networks or continuous state-space models. While traditional linear attention is usually position-unaware, where the recurrence order does not have an influence on the output, modern linear attention behaves more like recurrent neural networks (RNNs) with state-tracking and hidden memory. Therefore, these models explicitly incorporate recurrence, gating, or state dynamics to handle long sequences with linear complexity. Decay factor is the most important factor to bring the forgetting mechanism.

#### Data-independent decay

Retentive networks (RetNet)^3^ introduce a retention mechanism that replaces attention with a recurrent-style update using fixed decay coefficients. In a RetNet layer, each time step *t* maintains a state vector *s*_*t*_ that aggregates past inputs with exponential forgetting. The recurrence can be written as

(Equation 3) $$S_{t}=\gamma S_{t-1}+k_{t}^{⊤}v_{t}\text{,}$$

with *γ*∈(0,1) is a learned decay factor (per retention head) and $k_{t}^{⊤}v_{t}$ a contribution from the current token (*v*_*t*_ is a value projection of *x*_*t*_, and *k*_*t*_ is a key projection). The output is then obtained by a linear “query” projection: *o*_*t*_ = *q*_*t*_*S*_*t*_. Unrolling Equation 3 gives an explicit formula for retention:

(Equation 4) $$o_{t}=q_{t}S_{t}=\sum_{n=1}^{t}\gamma^{t-n}q_{t}k_{t}^{⊤}v_{t}\text{,}$$

which shows that contributions from token *n* are exponentially decayed by the factor *γ*^*t*-*n*^ by the time step *t*. Crucially, *γ* is a data-independent decay, which is a fixed parameter for the layer (often one per head in multi-head retention), not a function of the input content. It endows RetNet with an O(1) memory update like an RNN, while still allowing parallel computation during training via an equivalent matrix formulation. (For example, one can show that (3) is equivalent to a “retention matrix” form, Retention(*X*)=(*QK*^⊤^⊙*D*),*V*, where *D*_*t*,*n*_ = *γ*^*t*-*n*^ for *t* ≥ *n* implements the decay and causal masking.)

(1) RetNet’s retention mechanism shares themes with other data-independent recurrence models.
(2) Eagle^29^ improves RWKV design with outer-product memory, which is equivalent to linear attention. In RWKV series, the decay factor is parameterized as *γ* = *exp*(-*exp*(*w*)), where *w* is a data-independent learnable factor. In practice, both RetNet and Eagle achieve linear inference scaling with competitive performance, using fixed decays to forget old information. Empirically, RetNet uses a fixed scalar *γ* per head (often each layer has multiple retention heads with different *γ* values, giving a form of multi-scale decay), whereas Eagle uses a learnable scalar *w* to parameterize the decay factor.
(3) Lightning attention^45^^,^^46^ also proposes a linear attention layer augmented with a fixed scalar decay per head to achieve length-invariant computation speed. In lightning attention, the hidden state is essentially $s_{t}=\lambda s_{t-1}+k_{t}^{⊤}v_{t}$ for some constant *λ* (with *λ* learned or set by the model), which is in the same spirit as RetNet’s *γ* but optimized for hardware efficiency.
(4) H3^47^ introduces a recurrent state-space model^48^ into linear attention, using learned, data-independent exponential decay via state space models (SSM) for key-value outer-product hidden state. While linear attention supports efficient chunk-wise training, H3’s reliance on explicit state expansion for SSM computation imposes a practical bottleneck on the head dimension, thereby compromising its expressiveness.

In summary, data-independent decay methods maintain a persistent state that fades over time at a predetermined rate, enabling *O*(1) recurrence and constant memory per step. They sacrifice some adaptability, which motivates the introduction of data-dependent mechanisms in more recent models.

#### Data-dependent decay

While fixed decays offer simplicity and speed, they may under-utilize information from the input stream. Gated or data-dependent approaches make the forgetting factor itself a learned function of the current input. The general form of such a recurrent update is

(Equation 5) $$S_{t}=G_{t}S_{t-1}+k_{t}^{⊤}v_{t}\text{,}$$

where *S*_*t*-1_ is the previous state, and *G*_*t*_ is a gating tensor determined by the token *x*_*t*_. If *G*_*t*_ is close to 0 in some component, the past state in that component is largely forgotten at time *t*; if *G*_*t*_ ˜ 1, the past is retained. Unlike the constant *γ* in RetNet, here *G*_*t*_ varies with *t* via *x*_*t*_. Two notable examples of this strategy in LLM design are Mamba^4^^,^^49^ and gated linear attention (GLA).^5^

Mamba is a recurrent state-space model that endows the state decay rates with input dependence. In each Mamba layer, the base state evolution is similar to S4^48^ but the state matrix is effectively made dynamic. *G*_*t*_ is a group-wise vector ranging from 0 to 1 as a dynamic forget gate, which bridges a gap between attention and pure SSMs. Reported empirical results suggest that Mamba2 can achieve competitive or superior performance compared to transformers of similar or larger scale on specific language modeling tasks, reflecting the potential of data-dependent decay in long-sequence modeling.

GLA directly introduces a gating mechanism in the linear attention, where a gating function is embedded into a linearized attention layer to improve its expressiveness. GLA modifies retention recurrence by a learnable element-wise forget gate *G*_*t*_.

Beyond these, several other models similarly endow their recurrence with content-dependent gates:

(1) xLSTM^50^ replaces the standard sigmoid forget gate with an exponential transform of linear gate signals (with normalization), yielding a smooth, input-conditioned decay on its cell state.
(2) GateLoop^51^ applies a head-wise gate on retention, which enables a simple but effective data-dependent decay while maintaining efficient hardware implementation.
(3) HGRN^52^ introduces gated recurrence in the linear RNNs. HGRN2^53^ further adds state expansion into the HGRN framework. State expansion is equivalent to the key-value outer product in linear attention.
(4) Finch^29^ employs data-dependent gating on Eagle. Since Eagle is similar to retention with other orthogonal modifications, Finch also shows a deep connection with the above models.

In summary, data-dependent decay models augment linear attention or RNN-style architectures with content-based gates that control the flow of information. The results in the paper show that these models can often match or exceed transformer performance on language tasks, while scaling to very long inputs.

### Test-time optimization in linear attention

Beyond the efficiency gains offered by linear attention mechanisms, a significant advancement lies in their application to test-time optimization. Unlike fixed linear recurrence (e.g., constant or purely input-conditioned forgetting), this line of work treats state updates as explicit optimization steps at inference time, so memory is actively optimized rather than passively propagated. In Table 1, we demonstrate that all linear attention variants can be considered as the corresponding update rules.

**Table 1:** Update rule among different linear attention variants

Method	Update rule
Linear attention^2^	$S_{t}=S_{t-1}+k_{t}v_{t}^{⊤}$
RetNet^3^	$S_{t}=\gamma S_{t-1}+k_{t}v_{t}^{⊤}$
GLA^5^	$S_{t}=S_{t-1}\text{Diag}\left(a_{t}\right)+k_{t}v_{t}^{⊤}$
Mamba^4^	$S_{t}=\alpha_{t}S_{t-1}+b_{t}k_{t}v_{t}^{⊤}$
HGRN-2^53^	$S_{t}=S_{t-1}\text{Diag}\left(a_{t}\right)+\left(1-a_{t}\right)v_{t}^{⊤}$
DeltaNet^7^	$S_{t}=S_{t-1}\left(1-\beta_{t}k_{t}k_{t}^{⊤}\right)+\beta_{t}k_{t}v_{t}^{⊤}$
Gated DeltaNet^54^	$S_{t}=\alpha_{t}S_{t-1}\left(1-\beta_{t}k_{t}k_{t}^{⊤}\right)+\beta_{t}k_{t}v_{t}^{⊤}$
TTT^19^	$S_{t}=S_{t-1}-\beta_{t}\nabla_{S}l\left(S_{t-1};k_{t},v_{t}\right)$

Each model is a recurrence on matrix memory *S*_*t*_.

While large transformer models can exhibit prompt-conditioned adaptation by interpreting the prompt as a form of training data, recent innovations have integrated fast learning rules directly into the attention mechanism, effectively treating sequence processing as an online training process. FWP^6^ establishes a formal equivalence between existing linear attention mechanisms and fast weight learning. In the FWP paradigm, a slow neural network learns to program the “fast weights” of another network, often via additive outer products of self-invented key and value patterns. This section explores several models, including DeltaNet,^7^^,^^54^ Longhorn,^55^ test-time training (TTT) layers,^18^^,^^19^ Titans,^56^ and Atlas,^57^ that exemplify this paradigm of leveraging linear attention for test-time optimization through mechanisms like fast weight updates, viewed through a meta-learning lens.

#### Learning objective

From a meta-learning perspective, these models define an implicit learning objective that is optimized during inference. Denoting *q*_*t*_,*k*_*t*_,*v*_*t*_ as the query, key, and value at timestep t, the context memory *S*_*t*_ is optimized by the following objective:

(Equation 6) $$L_{t}\left(S\right)=\frac{1}{2}{\left\|f_{S}\left(k_{t}\right)-v_{t}\right\|}^{2}\text{.}$$

DeltaNet incorporates the classical delta rule,^6^ where *f*_*S*_(*k*_*t*_) = *Sk*_*t*_. Its update rule will be $S_{t}=S_{t-1}+\beta_{t}\left(v_{t}-S_{t-1}k_{t}\right)k_{t}^{⊤}$, which can be derived by minimizing the error between the current memory retrieval *S*_*t*-1_*k*_*t*_ and the new value *v*_*t*_. This signifies a step toward learning the key-value mapping online, effectively refining the memory based on the immediate context.

TTT^19^ generalizes the meta-learning objective with a different modeling architecture:

(Equation 7) $$f_{S}\left(k_{t}\right)=\left\{ \begin{array}{cc} \mathrm{LN}\left(S\;k_{t}\right)+k_{t}, & \text{TTT}-\text{Linear}\\ \mathrm{LN}\left({\text{MLP}}_{S}\left(k_{t}\right)\right)+k_{t}, & \text{TTT}-\text{MLP} \end{array}\right.\text{.}$$

The context network *f*_*S*_ enhances the capability of test-time meta-learning. However, since the gradient of *f*_*S*_ is much more complicated than a simple linear projection, the online update cannot be written as a simple rule.

#### Batch update

Batch update tries to solve the difficulties of the training parallelism when *f*_*S*_ works as a neural network. Usually, context memory is meta-learned with a batch size of 1, which is not feasible for general TTT models. Instead, analogous to chunk parallelism, TTT treats an entire chunk as a batch. There are no state updates within the batch (i.e., *S* remains consistent). After processing the batch, *S* is updated once using the aggregated gradients or update signals from all samples in the batch. This strategy preserves parallel efficiency while accommodating the training requirements of more complex architectures.

#### Momentum

Titans^56^ introduce momentum, which is commonly used in optimization, to strengthen the capability of the memory update mechanism:

(Equation 8) $$M_{t}=\left(1-\alpha_{t}\right)M_{t-1}+S_{t}\text{.}$$

$$o_{t}=q_{t}M_{t}\text{.}$$

The momentum term allows the memory to accumulate information gradually with an exponential moving average on the state *S*. This can be seen as a form of meta-learning where the update rule itself learns to be more stable and robust over long sequences.

#### Weight decay

Weight decay is another regularization technique in training, corresponding the forgetting mechanism within linear attention models. Gated DeltaNet^54^ and Titans employ weight decay in its memory update, serving as a learned forget gate to limit the influence of very old or noisy data. It corresponds to the selective state retention mechanisms found in architectures like RetNet^3^ and Mamba,^4^ where the decay mechanism is proven crucial for language modeling performance:

(Equation 9) $$S_{n}=\gamma_{n}S_{t-1}+\beta_{t}\left(v_{t}-S_{t-1}k_{t}\right)k_{t}^{⊤}\text{.}$$

In summary, these advancements in linear attention mechanisms are pushing the boundaries of test-time optimization by explicitly incorporating meta-learning principles into their architecture. Through fast weight updates, sophisticated memory management techniques, and online learning rules, these models are moving toward a paradigm where the distinction between training and inference becomes increasingly blurred, leading to more efficient and adaptable LLMs capable of learning and leveraging knowledge directly from the context.

### Discussion on other designs

#### Element-wise linear attention

Attention-free transformer^58^ leverages a simple weight $\exp\;\left(K_{t^{\prime }}+w_{t,t^{\prime }}\right)$ instead of *exp*(*QK*^⊤^):

(Equation 10) $$O_{t}=\sigma_{q}\left(Q_{t}\right)⊙\frac{\sum_{t^{\prime }=1}^{t}\;\exp\;\left(K_{t^{\prime }}+w_{t,t^{\prime }}\right)⊙V_{t^{\prime }}}{\sum_{t^{\prime }=1}^{t}\;\exp\;\left(K_{t^{\prime }}+w_{t,t^{\prime }}\right)}\text{,}$$

where $w_{t,t^{\prime }}$ is learned as pairwise position biases. Among the AFT variants, AFT-simple remove $w_{t,t^{\prime }}$ achieves linearized inference patterns. Since the product of *K* and *V* is element-wise, the recurrent state size is $R^{d}$ instead of outer-product state $R^{d\times d}$.

RWKV^59^ leverages the decay mechanism on AFT-simple. Specifically, RWKV improves AFT’s position biases with exponential decay *w*_*t*,*i*_ = −(*t*-*i*)*w*. The exponential formulation preserves the recurrence property while introducing the position biases.

Element-wise linear attention brings strong inference advantage. However, it suffers from the bottleneck of state size, under-performing matrix-based state size. Besides, even though element-wise memory is much faster than the outer-product memory, the end-to-end advantage is still marginal since other components occupy more than 95% latency^3^ with outer-product memory.

#### Multi-pass linear attention

Attention with bounded-memory control considers linear attention as a bounded memory module:

(Equation 11) $${\tilde{K}}_{n}=\sum_{i=1}^{n}K_{i}⊗\phi_{i},{\tilde{V}}_{n}=\sum_{i=1}^{n}V_{i}⊗\phi_{i}\text{.}$$

$$O_{n}=\text{softmax}\left(Q_{n}{\tilde{K}}_{n}^{⊤}\right){\tilde{V}}_{n}\text{,}$$

where ${\tilde{K}}_{n},{\tilde{V}}_{n}$ is the online-updated size-bounded keys and values. In implementation, ABC can be simplified as two-pass linear attention.

Gated slot attention (GSA)^60^ further introduces GLA into the ABC framework.^61^ Since ${\tilde{K}}_{n},{\tilde{V}}_{n}$ works as an implicit linear attention, GSA improves the update as a gated form:

(Equation 12) $${\tilde{K}}_{n}=\text{Diag}\left(\alpha_{n}\right){\tilde{K}}_{n-1}+\left(1-\alpha_{n}\right)⊗K_{n},{\tilde{V}}_{n}=\text{Diag}\left(\alpha_{n}\right){\tilde{V}}_{n-1}+\left(1-\alpha_{n}\right)⊗\text{.}$$

Multi-pass is an effective way to enhance linear attention’s expressive ability. However, it also brings additional computation overhead, which makes the architecture design as a trade-off between training efficiency and performance.

#### Bidirectional linear attention

Bidirectional attention plays an important role in encoder-style architectures such as BERT.^62^ The key difference in the linear formulation between unidirectional and bidirectional attention lies in the inference bottleneck and computational pattern. Encoder-only models typically exhibit *O*(*N*^2^) complexity. Moreover, each token in an encoder-only model has access to global information. As a result, bidirectional linear attention often maintains a constant-length global token pool to reduce complexity while preserving the use of the softmax function.

For instance, Linformer^63^ reduces the number of keys and values to a constant length through an additional matrix projection. Luna^64^ further extends the Linformer design by encoding the global token pool across model layers.

While bidirectional linear attention is effective for encoder-only architectures, these designs face significant challenges when applied to causal settings, as global-pool-based methods tend to be computationally expensive. Consequently, such architectures are not well-suited for LLMs.

### Hardware implementation

#### The parallel representation

We define the causal linear attention with gated decay as

(Equation 13) $$Q=\phi \left(XW_{Q}\right),K=\phi \left(XW_{K}\right),V=XW_{V},\gamma =f_{\gamma }\left(X\right)\text{.}$$

$$D_{nm}=\left\{ \begin{array}{cc} \prod_{i=m+1}^{n}\gamma_{i}, & n\geq m\\ 0, & n<m \end{array}\right.,O\left(X\right)=\mathrm{LN}\left(\left(QK^{⊺}⊙D\right)V\right)\text{,}$$

where $W_{Q},W_{K},W_{V}\in R^{d\times d}$, *f*_*γ*_ controls the sharpness of decay. The matrix $D\in R^{N\times N}$ encodes the causal mask with decay pattern, ensuring unidirectional flow of information.

When the decay is data-independent, *f*_*γ*_(·) = const∈(0,1]. Note that GroupNorm^65^ after linear attention is already a compulsory component,^3^ the explicit divisor of kernelized linear attention in Equation 2 is unessential.

The parallel representation is simple and easy to understand but has two shortcomings. First, the parallel form still preserves the *O*(*L*^2^) complexity, same as softmax attention. Second, it’s complexity increases when representing the ICL-style linear attention in section test-time optimization in linear attention.

#### The recurrent representation

**Figure 2:**
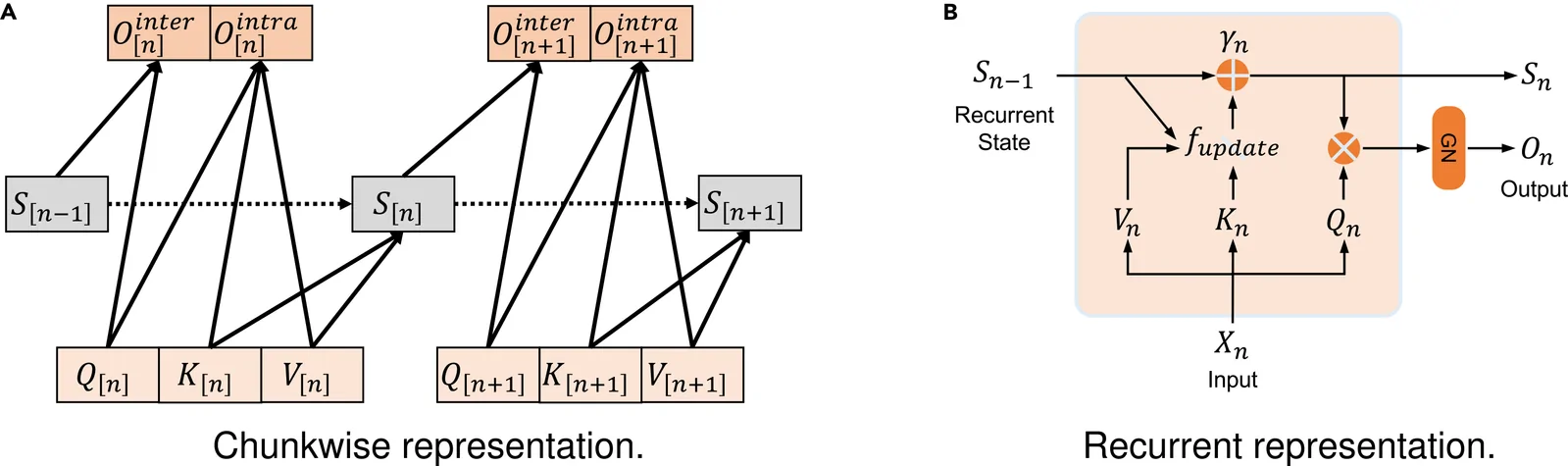
Dual form of linear attention

The parallel formulation above can be equivalently expressed in a recurrent form for stepwise decoding, as illustrated in Figure 2B. At each time step *n*, the output is computed as

(Equation 14) $$S_{n}=f_{\text{update}}\left(S_{n-1},K_{n},V_{n}\right),O_{n}=Q_{n}S_{n}\text{.}$$

$$f_{\text{update}}\left(S_{n-1},K_{n},V_{n}\right)=\left\{ \begin{array}{cc} \gamma_{n}S_{n-1}+K_{n}^{⊺}V_{n}, & \text{Linear}\;\text{attention}\;\text{with}\;\text{decay}\\ \gamma_{n}S_{n-1}+\beta_{n}\left(V_{n}-S_{n-1}K_{n}\right)K_{n}^{⊤}, & \text{ICL}-\text{style}\;\text{linear}\;\text{attention} \end{array}\right.\text{.}$$

This recurrent formulation enables efficient auto-regressive generation with constant memory by maintaining a single state vector *S*_*n*_.

While the recurrent representation reduces the computational complexity from *O*(*L*^2^) to *O*(*L*), it incurs substantial memory overhead during training. This is because *S*_*n*_ involves storing outer products of *K*_*n*_ and *V*_*n*_, which are prohibitively expensive for long sequences. As a result, the recurrent form is typically restricted to the decoding stage.

#### The chunkwise recurrent representation

The chunkwise representation combines the advantages of linear complexity and hardware-friendly parallelism.^3^^,^^66^ As shown in Figure 2A, taking decay-style linear attention as an example, given a chunk size *B*, let *x*_[*i*]_ denote the *i*-th chunk. Define the cumulative decay within a chunk as

(Equation 15) $$\beta_{\left(i-1\right)B+j}=\prod_{k=\left(i-1\right)B+1}^{\left(i-1\right)B+j}\gamma_{k},D_{\left[i\right]}\left(j,k\right)=\left\{ \begin{array}{cc} \frac{\beta_{\left(i-1\right)B+k}}{\beta_{\left(i-1\right)B+j}}, & j\leq k\\ 0, & \text{otherwise} \end{array}\right.\text{.}$$

The chunk-level memory state *R*_*i*_ is computed as

(Equation 16) $$R_{i}=K_{\left[i\right]}^{⊺}\left(V_{\left[i\right]}⊙\frac{\beta_{iB}}{\beta_{\left[i\right]}}\right)+\beta_{iB}R_{i-1}\text{,}$$

and the output for chunk *i* is given by

(Equation 17) $$O_{\left[i\right]}=\left(Q_{\left[i\right]}K_{\left[i\right]}^{⊺}⊙D_{\left[i\right]}\right)V_{\left[i\right]}+\left(Q_{\left[i\right]}R_{i-1}\right)⊙\beta_{\left[i\right]}\text{.}$$

This formulation offers a unified view of recurrence and parallelism: the first term captures intra-chunk dependencies, while the second term propagates inter-chunk memory through a single matrix-vector product. Owing to its efficiency and parallelizability, the chunkwise representation is typically adopted during the training and prefilling stages.

For ICL-style linear attention, hardware-friendly chunkwise representations have been developed using householder transformations.^7^^,^^54^ However, for more sophisticated variants, such as TTT and Titans, constructing an explicit chunkwise form remains challenging. Instead, these architectures typically rely on large batch sizes for memory updates, effectively simulating chunkwise computation through a fixed hyperparameter.

Kernel-level optimization is essential for achieving high performance. The widely adopted FLA^67^ provides a Triton-based implementation for many common linear attention modules. Alternatively, custom implementations in CUDA or TileLang^68^ are also provided by developer, which can be employed for further acceleration.

### Performance analysis

The experimental setup used for the evaluation of the models in this section is derived from the results reported in Gated DeltaNet,^54^ which extensively benchmarked various models across language modeling and retrieval tasks. The performance data presented here comes from the comparison of linear attention models against the transformer baseline, with a focus on both language modeling perplexity and retrieval accuracy. The models evaluated include RetNet, HGRN2, Mamba, Mamba2, DeltaNet, and Gated DeltaNet, as well as a standard transformer for comparison.

In terms of performance, as shown in Table 2, linear attention models have demonstrated the potential to outperform transformer-based models in language modeling perplexity. Models such as RetNet, Mamba, and Gated DeltaNet achieve lower perplexity compared to the transformer baseline, indicating that linear attention mechanisms can lead to more efficient language models while maintaining or improving performance in language modeling tasks.

**Table 2:** Linear attention models showing potential to outperform transformer in language modeling

Model	Wiki. ppl ↓	LMB. ppl ↓
Transformer	18.53	18.32
RetNet	19.08	17.27
HGRN2	19.10	17.69
Mamba	17.92	15.06
Mamba2	16.56	12.56
DeltaNet	17.71	16.88
Gated DeltaNet	16.42	12.17

On the retrieval side, as shown in Table 3, linear attention models have made significant improvements in retrieval accuracy, approaching the performance of transformer-based models. For instance, gated DeltaNet achieves competitive retrieval performance on tasks, such as SWDE, SQD, and FDA, when compared to the transformer. These improvements suggest that while linear attention models are still catching up to transformer models in retrieval tasks, they show great promise in bridging the gap.

**Table 3:** Recall-world retrieval accuracy

Model	SWDE	SQD	FDA	TQA	NQ	Drop	Avg
Transformer	29.5	38.0	52.2	58.3	22.5	21.6	37.0
RetNet	14.0	28.5	7.0	54.4	16.2	17.3	22.9
HGRN2	8.3	25.3	4.8	51.2	14.2	16.9	20.1
Mamba	9.8	25.8	3.7	54.3	14.9	17.4	21.0
Mamba2	19.1	33.6	25.3	61.0	20.8	19.2	29.8
DeltaNet	17.9	30.9	18.4	53.9	17.3	18.6	26.2
Gated DeltaNet	25.4	34.8	23.7	60.0	20.0	19.8	30.6

Linear attention improvements approaching transformer retrieval performance.

Regarding efficiency, it is worth noting that the inference speeds of these linear attention models are quite similar. This similarity arises from the fact that the KV cache memory access patterns are generally aligned across these models. As a result, their inference speeds are nearly identical, with no significant performance difference observed in handling memory access. Therefore, the primary differences between these models lie in their performance on language modeling and retrieval tasks, as discussed above.

## Sparse attention

Sparse attention methods employ the inherent sparse property in attention computation and approximate full attention by

(Equation 18) $$\text{Attn}\left(Q,K,V\right)=\text{softmax}\left(QK_{\left[S\right]}^{⊤}\right)V_{\left[S\right]}\text{,}$$

where S(t) is a subset of indices that query vector *Q*(*t*) attend to. Different methods design different selection criteria for S(t), taking both selection accuracy and hardware efficiency into consideration. Reduce into sub-linear or linear complexity for prefilling or fixed budget for decoding.

Conceptually, sparse attention differs from linear attention in two key dimensions. From a computational perspective, sparse attention does not inherently reduce memory footprint, as it still requires storing the full KV cache. Instead, it primarily focuses on accelerating the generation phase by reducing the number of attended tokens. From a performance standpoint, while linear attention often involves lossy compression of the global context into a fixed-size state, sparse attention maintains the original resolution of selected tokens, allowing its theoretical upper bound to closely approach that of full attention.

### Fixed-pattern sparse attention

Fixed-pattern methods represent the earliest and most direct approach within the sparse attention paradigm. Because these methods rely on static, pre-defined masks rather than data-dependent routing, they offer the highest degree of hardware friendliness and are the easiest to optimize at the infrastructure level. By leveraging predictable memory access patterns, fixed-pattern designs were the first to be successfully integrated into early long-context transformers and modern system-level kernels.

#### Local window attention

Local-window attention confines each query to interact only with neighboring tokens inside a fixed sliding window *w*, thus lowering both memory and compute while preserving local context.

Sparse transformer^8^ first applies local-window (row) attention, where *w* is close to $\sqrt{N}$, then augments it with an extra column attention that summarizes previous locations and propagates information globally. **GPT-3**^32^ also adopts a sparse attention pattern similar to that used in the sparse transformer.

StreamingLLM^21^ found that a large amount of attention score is allocated to initial tokens in the input sequence, which they refer as “attention sink.” They propose a simple fixed-pattern attention, which keeps only the sink tokens and sliding window tokens. For instance, given an input sequence with length *n*, selected token subset S(t) for query token *q*_*t*_ in StreamingLLM is formulated as

(Equation 19) S(t)={j∣0≤j≤s∨t−w≤j≤t},∀t∈[1,n],

where *s* is the sink token size and *w* is the sliding window size. For better hardware efficiency, StreamingLLM with block granularity^69^ keeps sink tokens and local tokens in a block-wise manner, enabling efficient memory loading and computation.

#### Dilated attention

LongNet^22^ introduces dilated attention as a fixed sparse pattern for long context training and inference. Dilated attention expands the attention field exponentially as the distance grows, thereby reducing the complexity of attention from *O*(*L*^2^) to *O*(*L*). Specifically, after dividing the input along sequence dimension into segments, with length *w*, dilated sparse index is selected from each segment with an interval *r*. The selected indices of segment *i* are

(Equation 20) $$\hat{I_{i}}=\left[iw,iw+r,iw+2r,\ldots ,\left(i+1\right)w-1\right]\text{.}$$

Sparsified segments $Q_{\hat{I_{i}}},K_{\hat{I_{i}}},V_{\hat{I_{i}}},i\in \left\{0,1,\ldots ,\frac{n}{w}\right\}$ are fed into the attention in parallel, getting attention output *O*. Combining the attention output of different segment sizes and dilation rates ${\left\{r_{i},w_{i}\right\}}^{k}$, the final attention is computed as

(Equation 21) $$O=\sum_{i=1}^{K}\alpha_{i}O{|}_{r_{i},w_{i}},\alpha_{i}=\frac{s_{i}}{\sum_{j}s_{j}}\text{,}$$

where *s*_*i*_ denotes the denominator of the attention softmax for $O{|}_{r_{i},w_{i}}$. **LogSparse**^70^ adopts an exponentially sparse attention scheme in which each position attends to only *logN* tokens, which can be seen as an instance of exponentially dilated attention.

### Dynamic sparse attention

**Figure 3:**
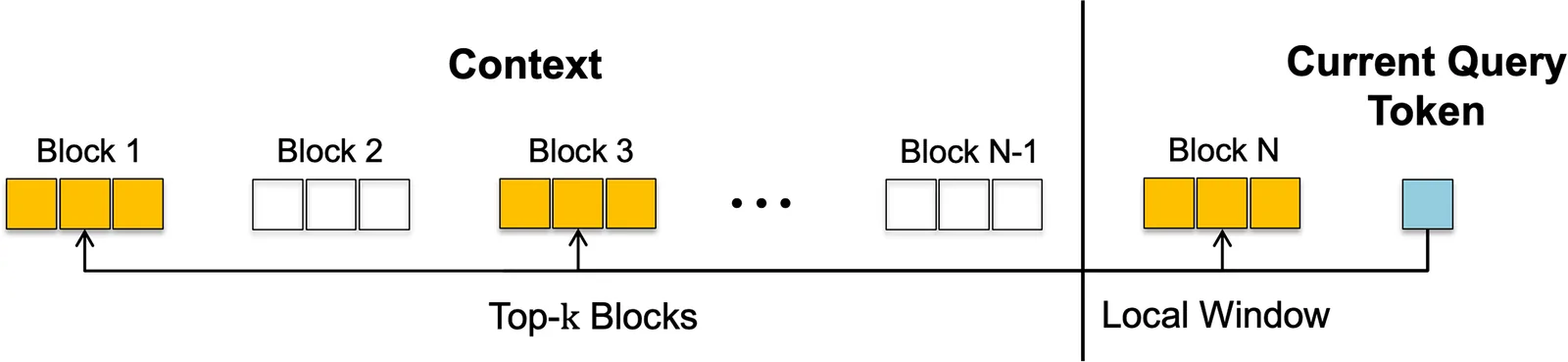
Block-sparse attention: The long sequence is divided into several blocks, and each token attends only to its local window and the top-k related blocks

The shift from fixed-pattern to dynamic sparse attention addresses the inherent rigidity of static masks, which fail to capture the dynamic, data-dependent nature of attention in complex tasks. Within this category, block-sparse and token-sparse methods present different trade-offs. Block-sparse attention is more hardware-friendly, as its structured sparsity preserves block-aligned memory access and maps well to efficient GPU kernels, making it suitable for accelerating both prefill and decode. Its main limitation is that the coarser sparsity pattern is harder to make lossless, so performance depends on accurately identifying important blocks. In contrast, token-sparse attention offers finer-grained flexibility and can better approximate full attention, especially during decoding, but its irregular memory access and selection overhead make prefill acceleration difficult in practice. Generally, given an input sequence with length *n* and a block size *b*, we could divide $Q,K,V\in R^{n\times d}$ each into $\frac{n}{b}$ blocks, each block sized *b*×*d*. The goal is to approximate a block-level mask *M*∈{0,1}^*n*/*b*×*n*/*b*^ used for selecting critical blocks for computation, as illustrated in Figure 3.

(Equation 22) $$\text{Attn}{\left(Q,K,V\right)}_{i}=\sum_{j=1}^{n/b}M_{ij}\cdot \text{softmax}\left(Q_{i}K_{j}^{T}\right)V_{j}\text{,}$$

where token-sparse attention corresponds to *d* = 1. For block-sparse attention, block-wise selection is crucial to achieve efficient computation on modern GPUs.

#### Block-sparse attention for prefill

Methods that use block-sparse attention for prefilling approximate top-k blocks cover the majority of attention score with high recall ratio, thus reducing the computation complexity of attention from *O*(*L*^2^) to *O*(*LK*). Given the standard attention weights function

(Equation 23) $$S=\text{softmax}\left(QK^{T}-c\left(1-M\right)\right)\text{.}$$

$$\min\left|S\left(M\right)-S_{dense}\right|\text{,}$$

where *M* is our block-wise sparse mask defined as above, and *c* is a large constant, such as 1*e*5, ensuring that less important attention weights approach zero after the softmax computation. The objective of the block-sparse attention is to achieve greater speedup with minimal overhead while retaining as much of the attention weights as possible. MInference^9^ observed that there are three patterns in attention weights: streaming (A shape) pattern, vertical-slash pattern, and block-sparse pattern. It determines the optimal pattern for each attention head offline and dynamically builds sparse indices based on the assigned pattern during inference. FlexPrefill^71^ proposes a context-aware sparse attention mechanism that dynamically adjusts attention patterns and computational budgets in real-time. XAttention^23^ presents a block-sparse attention framework that utilizes antidiagonal scoring to predict the importance of attention blocks, which could efficiently identify and prune non-essential blocks, achieving high sparsity and substantial computational gains. SpargeAttn^72^ also employs block-level sparse attention for prefill, which is done through a double-stage online filtering process: the first stage rapidly predicts the attention map to skip certain matrix multiplications, and the second stage applies a softmax-aware filter to further eliminate unnecessary computations.

#### Block-sparse attention for decode

Methods that employ block-sparse attention for decoding dynamically select a subset *S* of *K*,*V* vector that contain the most critical tokens for each decoding step, thus reducing memory loading and improving efficiency. **Quest**^10^ approximates the criticality of each block by calculating an upper bound of attention weights. For block *K*_*i*_, we maintain element-wise Min and Max Key *m*_*i*_ and *M*_*i*_, respectively, through

(Equation 24) $$m_{i,d}=\min\left(K_{i,d}\right),M_{i,d}=\max\left(K_{i,d}\right)\text{,}$$

where *min*(·) and *max*(·) are applied element wise for each dimension *d*. Given query *q*, the approximate attention score for block *i* is given by

(Equation 25) $$score_{i}=\sum_{j=1}^{d}\max\left(q_{j}\times M_{i,j},q_{j}\times m_{i,j}\right)\text{.}$$

Then it selects top-k blocks with the highest *score* as sparse subset *S* for attention calculation.

(Equation 26) S=argtopk(score,k).

DoubleSparsity^73^ approximates critical tokens efficiently through reducing the matrix multiplication dimension of calculating *QK*^*T*^ product. It first offline calculates outlier channels in *QK*^*T*^, denoted as *C*. Then it selects top-k tokens with the ighest approximate attention score $\hat{s}$ as the sparse subset *S*.

(Equation 27) $$Q_{label}=Q_{\left[C\right]},\hat{s}=Q_{label}K_{label}^{T},S=\text{argtopk}\left(\hat{s},k\right)\text{,}$$

ReSA^74^ combines training-free block-sparse estimation and GQA sharing, contributing to better efficiency. Besides, ReSA proposes a rectification stage to control the KV cache accumulation error. ReSA shows an advantage on long-sequence generation tasks.

#### Training-aware sparse attention

Training-aware sparse attention learns block- or token-level selection during pretraining or adaptation via auxiliary gating networks (often trained with self-distillation from dense attention), rather than relying only on training-free heuristics at inference time. We first review training-aware block-sparse attention, then token-sparse variants that refine granularity or amortize selection across layers.

#### Training-aware block-sparse attention

Landmark^75^ proposes using special landmark tokens to represent each block and trains the attention mechanism to directly retrieve top-k blocks via these landmark tokens. However, it did not experiment on large-scale pretrained models. SeerAttention^24^^,^^76^ trains the gating network on pretrained LLMs in a self-distillation manner. To obtain the importance score for each block, it first conducts pooling to *Q* and *K* along the sequence dimension, denoted as *P*_*k*_ and *P*_*q*_. The downsampled *Q*,*K* are then passed through a learnable linear layer *W*_*q*_ and *W*_*k*_. Matrix multiplied results of projected *W*_*q*_*P*_*q*_(*Q*) and (*W*_*k*_*P*_*k*_(*K*)) go through the softmax operator as a gating process:

(Equation 28) $$score=\text{softmax}\left(\left(W_{q}P_{q}\left(Q\right)\right)\cdot \left(W_{k}P_{k}\left(K\right)\right)\right)\text{.}$$

The learnable linear layers are trained to align with the 2D maxpooled results of the original LLM through a self-distillation manner. The distillation loss is computed as

$$gt=\text{MaxPool}2D\left(\text{softmax}\left(QK^{T}\right)\right),loss=D_{KL}\left(\;gt\|score\;\right)\text{.}$$

During inference, the gating score is used to predict the block-level sparsity through top-k or thresholding for sparse computation and efficiency. MoBA^12^ integrates trainable sparse attention into the pretraining stage. It proposes a mixture of block attention, which applies the top-k mechanism from MoE as the gating mechanism to decide critical blocks for each query token. The importance score of each block is computed by the inner product between query token *q* and the mean pooling result of block *K*_*i*_ along the token dimension:

(Equation 29) $$s_{i}=<q,P_{mean}\left(K_{i}\right)>\text{.}$$

Then the top-k blocks with the highest *s* score are selected for *q* in computing attention. Notably, the top-k block selection used by MoBA is not differentiable. Therefore, the sparsity pattern is still estimated in a training-free pattern in the pretraining stage, enabling both efficient inference and accelerated training. **NSA**^11^ introduces a training-aware mix-granularity sparse attention mechanism consisting of three branches *C*∈{cmp,slc,win}, which correspond to compression, selection, and sliding window strategies, respectively. NSA leverages a differentiable compression branch to learn the block selection score. Combining the three branches, the attention output of NSA is given by

(Equation 30) $$o=\sum_{c\in C}g^{c}\cdot \text{Attn}\left(q,K^{c},V^{c}\right),g_{c}\in \left[0,1\right]\text{.}$$

For the compression branch *c* = cmp, block *i*’s key $K_{i}\in R^{d_{k}\times b}$ is compressed into a single key $K_{i}^{\text{cmp}}\in R^{d_{k}\times 1}$ through a learnable MLP layer *φ*. For the selection branch *c* = slc, top-k block is selected based on bthe lock importance score *p*, which could be directly obtained from the compression branch. **InfLLM-v2**^31^ adopts a training-aware top-k block sparse attention mechanism similar to MoBA. For top-K block selection accuracy, it divides the blocks into small-granularity kernels with overlap and performs aggregation on kernel importance scores within each block. **HySparse**^77^ proposes a hybrid sparse attention architecture with oracle token selection and KV cache sharing. It first uses a vanilla full attention layers to select oracle blocks and then uses them for deeper N layers of block-sparse attention.

#### Training-aware token-sparse attention

DSA^78^ inherits the self-distillation training pipeline from SeerAttention. However, to further alleviate the performance damage, DSA makes the sparsity computation on the token-level instead of block-level. Since the score computation of token-level sparsity is relatively high, DSA leverages a smaller head dimension and lower precision to reduce the cost. IndexCache^79^ proposes cross-layer index reuse to amortize the cost of index selection. It extends the indexer KL loss to multiple layers, reducing the cost of DSA without compromising performance.

#### System-level design choices

Learning-aware sparse attention^11^^,^^12^^,^^31^ begins to take kernel implementation and efficient execution into consideration. For real wall-time acceleration of dynamic sparse attention, FlashAttention^28^ is used for attention computation in an efficient tilling mechanism, introducing requirements and opportunities for better utilization of hardware resources, including the following.

(1) To avoid inconsistency in memory access for prefilling, in SeerAttention^24^ and MInference,^9^ block size *b* is typically set to a relatively large value of at least 64.
(2) To align with the minimal requirement of grouped matrix multiplication instruction on GPU tensor cores, in NSA^11^ and InfLLM-v2,^31^ the number of K and V heads within a query group is set to at least 16.
(3) To reduce memory access, NSA^11^ and InfLLM-v2^31^ forces share selected blocks among query groups, which is done through conducting pooling on block-level importance score within query groups.
(4) To amortize the cost of index selection, IndexCache^79^ extends the indexer KL loss to multiple layers, reducing the cost of DSA without compromising performance.

### Clustering attention

The transition from block-level selection to clustering-based methods is motivated by the fact that tokens adjacent in position do not necessarily share semantic relevance. While block-wise partitioning respects hardware-friendly physical layout, it often fails to align with the underlying attention patterns, which are driven by content similarity rather than spatial proximity. By shifting the focus from spatial blocks to feature-space grouping, clustering attention provides a superior theoretical upper bound for performance, as it ensures that the most semantically critical tokens are captured regardless of their distance in the original sequence. RetrievalAttention^80^ employs approximate nearest neighbor search (ANNS) for selecting critical K clusters. To address the challenge of the out-of-distribution nature between the query and key vectors in the attention mechanism, it introduces an attention-aware vector search algorithm that adapts to the distribution of query vectors. ClusterKV^27^ selects tokens at the granularity of semantic clusters, overcoming the issue of internal fragmentation of page-level retrieval methods such as Quest. After the prefilling stage, tokens are clustered through *k*-means algorithm. The semantic similarity between token *i* and *j* are measured through the cosine similarity of key vectors $D\left(i,j\right)=1-\frac{<k_{i},k_{j}>}{|k_{i}\left|\cdot \right|k_{j}}$. Semantic clusters are represented by their centroids $\mu_{1},\mu_{2},\ldots ,\mu_{C}\in R^{d}$. At each decoding step, clusters are selected based on the ranking of query token *q* and centroids *μ*_*i*_’s attention weights, i.e., $q\mu_{i}^{T}$. MagicPIG^26^ leverages locality sensitive hashing (LSH) sampling to efficiently approximate attention computation. It employs LSH to map similar query and key vectors to the same hash buckets and offloads storage and partial computation to the CPU to address the KV cache bottleneck. It also introduces Oracle top-k sampling as a better strategy than brute force top-k.

### Bidirectional sparse attention

Bidirectional sparse attention builds upon encoder-style architecture, using static pattern or block-level sparsity to accelerate attention computation. Block sparsity is widely used in bidirectional sparse attention. BigBird^81^ uses block-wise random attention, which acts as a bridge to shorten indirect paths between tokens. Longformer^20^ uses static global-local hybrid attention. It also relies on block-level sparsity with additional global and random links, facilitating structured computation and memory-efficient parallelism. Clustering-based methods are also used in bidirectional sparse attention. Reformer^25^ uses LSH to assign similar tokens to the same bucket. Routing transformer^82^ performs online *k*-means clustering per layer. ClusterFormer^83^ introduces a differentiable clustering module co-trained with downstream objectives. These methods reduce computation by grouping related tokens while maintaining performance through learned adaptability.

### Efficiency analysis

#### Sparse attention for prefill

To provide a coarse quantitative view of sparse-attention prefill efficiency, we summarize the reported attention speedup of several representative methods in Table 4. Since the numbers are collected from different papers, model scales, and implementations, they should be interpreted as an approximate comparison of practical trends rather than a strictly controlled benchmark. For each method, we use the best reported result from the corresponding paper. Most existing sparse prefill methods are either training-free or rely on lightweight fine-tuning, and their practical performance largely hinges on the ability to efficiently locate the most important attention blocks without introducing excessive search overhead. Therefore, the key to obtaining high realized speedup is not merely imposing sparsity but designing better block-scanning and block-selection algorithms that can preserve the behavior of full attention as much as possible while reducing the amount of computation.

**Table 4:** Attention prefill speedup of sparse attention methods relative to full attention

SeqLen	8K	16K	32K	64K	128K	256K
MInference	0.2×	0.4×	0.8×	1.1×	2.5×	4.2×
SeerAttention	1.6×	–	1.7×	–	1.5×	–
FlexPrefill	0.4×	1.1×	2.2×	3.4×	4.8×	6.2×
XAttention	1.7×	2.2×	3.2×	4.3×	7.1×	9.8×

XAttention is reported with *S* = 8. For each method, the values are taken from the best reported results from related papers.

#### Sparse attention for decode

**Figure 4:**
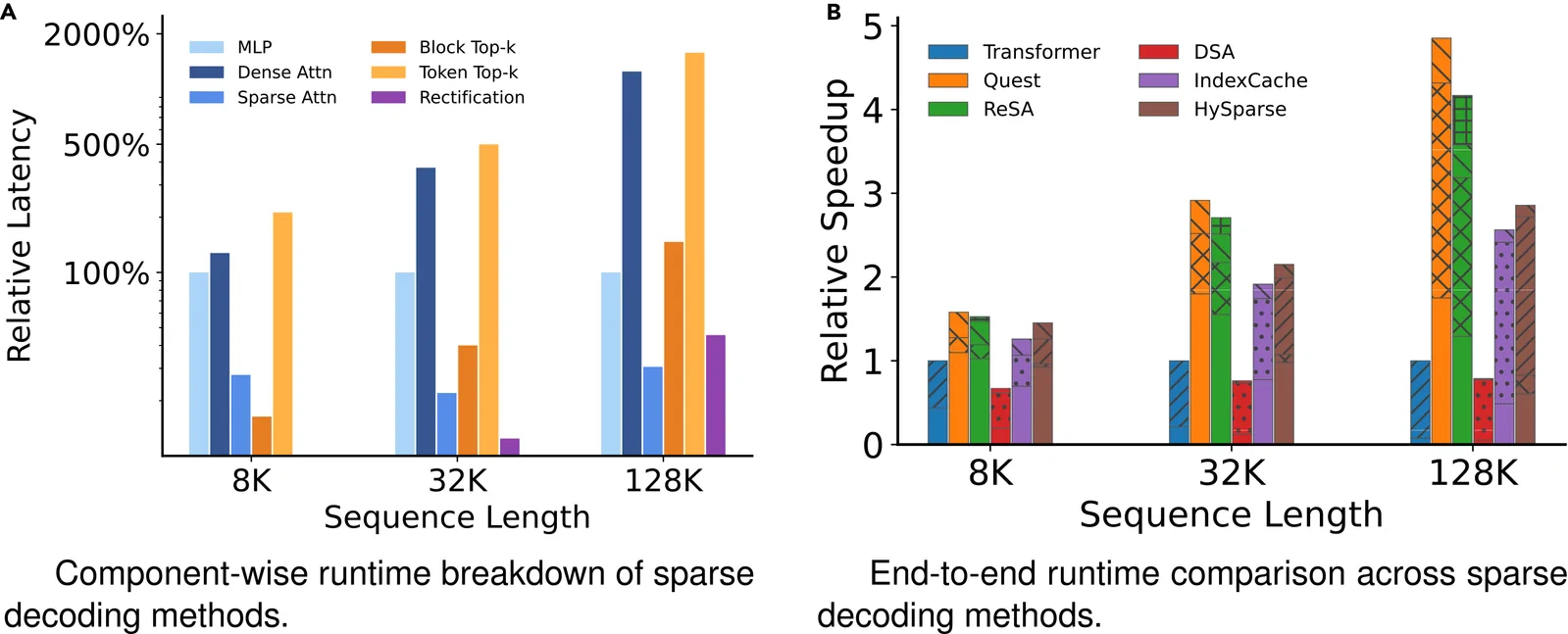
Efficiency breakdown of sparse decoding acceleration methods Token-level top-K methods may offer stronger sparsification flexibility but their realized speedup is often limited by the overhead of index selection. Practical acceleration is more likely to come from structured block-sparse execution or from amortizing top-K overhead.

To complement the prefill results, we further compare several sparse decoding acceleration methods using our own measurements on NVIDIA B200 GPUs. The results are shown in Figure 4. First, although token-level top-K sparsification generally has a higher potential upper bound in terms of approximation quality, its realized speedup is often limited in practice. In particular, on newer GPUs such as B200, operators dominated by CUDA-core-based top-K selection do not benefit as much as tensor-core-based matrix multiplication kernels, making their relative speedup increasingly modest. For example, DSA provides little end-to-end speedup in our measurements despite reducing the attention computation itself. Second, achieving practical decoding acceleration typically requires either improving the effectiveness of block-sparse execution, so that more work is shifted to efficient structured kernels, or amortizing the overhead of top-K selection, for example, by sharing sparse indices across layers. These results again highlight that, for sparse decoding, the main challenge is not only reducing arithmetic complexity but also ensuring that the resulting computation maps efficiently to modern GPU hardware.

## Pretrained LLM with efficient attention

### Pretrained models with uniform efficient attention

While early explorations of linear attention were often confined to smaller-scale models, recent advancements have demonstrated their successful scalability to the multi-billion parameter range, establishing them as viable and highly efficient alternatives to the standard transformer. These models, built purely on linear attention or its architectural equivalents like state-space models (SSMs) and RNNs, retain their signature inference efficiency even at large scales.

#### RWKV-based model

The RWKV project represents a sustained and influential effort to create a scalable RNN architecture that combines the parallelizable training of transformers with the efficient inference of traditional RNNs.^59^ For instance, the Eagle (RWKV-5) series introduced matrix-valued states to increase capacity, while subsequent iterations like Finch (RWKV-6)^29^ and Goose (RWKV-7)^84^ incorporated dynamic recurrence and expressive state evolution mechanisms (e.g., a delta-rule) to enable more complex, data-dependent state transitions.

#### Mamba-based model

The success of the Mamba architecture,^4^ with its data-dependent selection mechanism (section data-dependent decay), has spurred a wave of adoption and scaling initiatives from major research labs. Falcon Mamba^30^ is based on the pure Mamba-based architecture to demonstrate performance competitive with leading transformer models on a wide range of general-purpose language benchmarks, validating the architecture’s viability for such tasks while retaining its signature constant-time inference. Further evidence of this paradigm’s potential is provided by Codestral Mamba,^85^ built on the Mamba-2 architecture. While specialized for code generation, it achieves state-of-the-art results on relevant benchmarks and supports a 256k token context, demonstrating the scalability and effectiveness of the SSM approach within a complex and structured domain.

#### Sparse-based model

MiniCPM-4^31^ introduces a two-stage sparse attention mechanism that dynamically selects relevant key-value blocks for each query token based on semantic similarity. MiniCPM-4 leverages InfLLM-v2, a block sparse attention variant to replace the standard attention mechanism. Moreover, a lightweight LogSumExp approximation enables efficient top-k selection, making the method scalable to extremely long sequences. Together, these techniques allow MiniCPM-4 to balance fine-grained contextual awareness with tractable memory and compute requirements, making it a strong candidate for long-context modeling.

### Pretrained models with hybrid efficient attention

**Figure 5:**
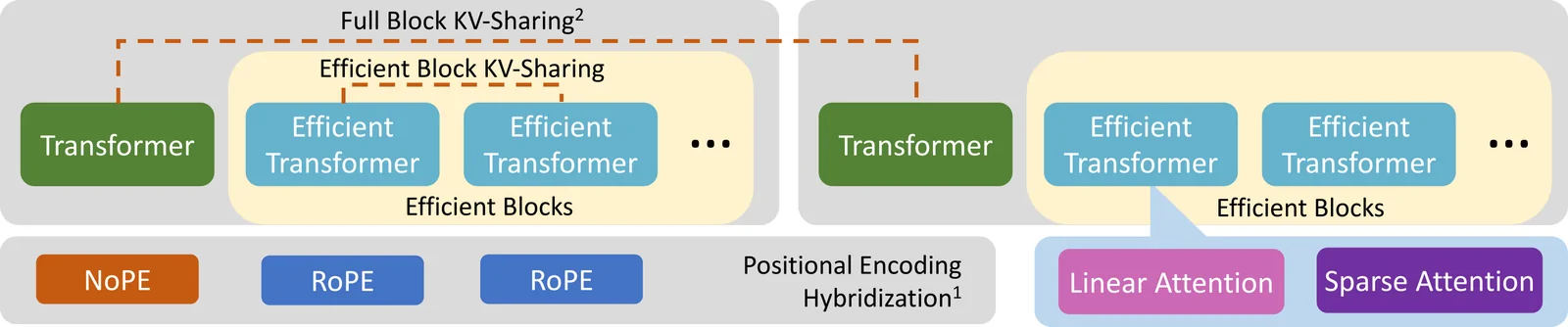
Representative stacking patterns of hybrid efficient attention in pretrained LLMs Superscript ^1^ marks models that mix different positional encoding schemes across layer types, and ^2^ marks KV-sharing designs that reuse key-value states across multiple layers.

With the increasing demand for efficient long-context modeling and diverse paradigms, recent research has extensively explored hybrid attention mechanisms. Such strategies combine global and local attention components, often interleaving specialized layers to balance computational cost and performance. Representative stacking patterns of these hybrid designs are illustrated in Figure 5. Mechanistically, a plausible interpretation is that the inserted full-attention layers act as expensive retrieval anchors, periodically recovering information that may be compressed or lost in local layers. This perspective clarifies why hybridity is effective in practice and why it is better viewed as a pragmatic compromise for near-lossless quality-efficiency trade-offs, rather than a conceptual replacement for linear models.

#### Sparse hybrid model

GPT-3^32^ integrates a hybrid attention mechanism by interleaving dense and locally banded sparse attention layers, inspired by the sparse transformer.^8^ Dense attention provides full-context modeling, while sparse layers adopt fixed or strided patterns to reduce the number of attended tokens. This design enables GPT-3 to efficiently scale to large model sizes using a fixed context window of 2,048 tokens, balancing modeling capacity and computational efficiency.

#### Linear-full hybrid model

Jamba^36^ and MiniMax-01^35^ combine linear and full attention layers to achieve an efficient trade-off between throughput and expressiveness. MiniMax-01 employs lightning attention across most layers, inserting softmax-based full attention every eight layers. Jamba adopts a similar ratio, inserting one transformer layer into every eight-layer Mamba block. Both achieve faster decoding and improved long-sequence performance by limiting the use of computationally intensive full attention.

#### Local-full hybrid model

Gemma 3,^33^ Command A,^38^ and LLaMA-4-Maverick^34^ alternate between local and global attention layers, with a shared design philosophy of using global layers sparsely, e.g., every 4–6 layers, to enhance efficiency. While local layers adopt sliding-window patterns, the key difference lies in position encoding strategies. Gemma 3 modulates RoPE base frequencies—assigning 10K for local and 1M for global layers—to better capture long-range dependencies. Command A and LLaMA-4-Maverick mixes RoPE-based local layers with full attention layers that omit positional embeddings entirely, allowing stronger long sequence performance.

#### Advanced hybrid model

Character.AI^86^ interleaves local attention with sliding windows and sparse global attention layers applied every six layers. Especially, they reuse the global attention layer’s key-value representations across multiple non-adjacent layers. This KV sharing mechanism enables efficient long-context processing with reduced memory and latency overhead. YOCO^37^ and Phi-4-mini-flash^87^ adopt a dual-decoder architecture that separates the prefill and generation phases. The self-decoder utilizes linear attention mechanisms such as RetNet and sliding-window attention for both prefill and generation, while the cross-decoder is activated only during generation. A single-layer global KV cache is used throughout, allowing linear-time prefill and efficient decoding with minimal GPU memory consumption. In summary, these recent advances underscore the trend toward hybridizing attention mechanisms to achieve balanced performance across varying computational constraints and sequence lengths. Each architecture uniquely contributes insights into effectively combining local detail management with global context integration, thereby providing valuable frameworks for future attention mechanism developments.

### Takeaways

Rather than treating efficient attention mechanisms as isolated alternatives, current research increasingly points to a small set of design principles for achieving near-lossless acceleration. Below, we summarize the most consistent conclusions that emerge from recent algorithmic and systems developments.

### Hybridity is the most reliable path

There is a growing consensus that while purely linear attention architectures offer impressive inference speeds, they frequently incur a non-trivial performance penalty in complex reasoning and ultra-long context retrieval. Consequently, hybrid models have emerged as the preferred architecture. These designs not only maintain the near-lossless performance of standard transformers but also demonstrate the potential to exceed them by balancing global associative memory with precise local retrieval.

### Sparse attention struggles with optimization and selection quality

Training sparse attention models from scratch remains significantly more challenging than dense counterparts. This performance gap primarily stems from gradient sparsity, where restricted connectivity leads to suboptimal optimization dynamics and lower learning efficiency. While self-distillation has shown promise in bridging this gap, it remains an auxiliary solution, indicating that sparse-from-scratch strategies require further foundational innovation.

### Linear and sparse mechanisms play complementary roles

From a unified functional perspective, linear and sparse mechanisms are increasingly viewed as complementary rather than competitive. In a modern efficient stack, linear attention serves as an effective state compressor for fast inference and KV cache reduction, while sparse attention acts as a high-fidelity module to replace full attention during decoding. A robust architecture leverages this synergy to navigate the pareto frontier of computational throughput and predictive accuracy.

### Hardware-algorithm co-design determines realized gains

A recurring takeaway is that theoretical FLOPs reduction does not inherently translate to wall-clock speedups. Modern efficient attention is inextricably bound to hardware optimization. For instance, the utility of linear attention hinges on switching between recurrent and parallel forms based on the workload, while sparse attention’s success depends on resolving the tension between theoretical sparsity and the massive parallelism required by modern GPUs. Taken together, these observations suggest that, in general-purpose long-context language models, efficiency should not be pursued by accepting avoidable quality degradation. Instead, the central question is how to realize near-lossless acceleration, and current evidence points to a few recurring ingredients: hybrid designs, better sparse optimization and selection, complementary linear and sparse components, and careful hardware-algorithm co-design.

## Outlook

This survey presents a comprehensive overview of efficient attention mechanisms, focusing on their algorithmic foundations, practical implementations, and integration into large-scale pre-trained language models. By categorizing linear and sparse attention into well-defined paradigms, we identify key design principles that enable scalability, computational efficiency, and long-context capability. We also analyze how these mechanisms are deployed in the state-of-the-art models, either as standalone architectures or as part of hybrid designs that balance local and global computation. Looking forward, we highlight several key directions that are expected to shape future research in this area.

### Architectural understanding of hybrid models

While prior work in linear attention has largely focused on standalone linear architectures, hybrid models are often constructed by combining off-the-shelf linear attention modules with dense or local components. However, it remains unclear whether a stronger linear backbone directly translates to improved hybrid performance. Future work should investigate hybrid models as a distinct architectural class, seeking to understand their composition, interaction effects, and optimization dynamics.

### Lossless sparse attention and extended context

Sparse attention remains challenged by a trade-off between accuracy and computational gain. Fully trained sparse models often underperform dense ones, while post-training sparse approximations face limitations due to a lack of end-to-end training. A major research frontier lies in developing sparse attention mechanisms that maintain the expressiveness and accuracy of dense attention, while scaling to much longer contexts. Additionally, the relationship between sparse budget and context length is poorly understood, where fixed top-k schemes may degrade with longer sequences, calling for more adaptive strategies.

### Mechanistic insights into sparse and hybrid attention

While empirical studies have repeatedly demonstrated that hybrid attention models can match or exceed dense models using fewer attention computations, the underlying reasons for this effectiveness remain insufficiently explored. Besides, it is especially important to investigate whether the sparsity patterns that work well in synthetic benchmarks generalize to real-world tasks, and to characterize the limits of sparsity-based generalization.

### Broader opportunities for efficient architectures

Efficient attention architectures are also valuable beyond lossless long-context modeling. They can serve as high-throughput draft models in speculative decoding, as compact latent modules for compression and reconstruction, as context summarization components in retrieval pipelines, and as practical solutions for on-device or low-latency deployment under strict memory and throughput constraints. Looking forward, an important direction is to co-design these architectures with the objectives of specific applications, rather than evaluating them solely by how closely they match dense attention on long-context tasks. As attention-based models continue to evolve, we expect further convergence between architectural innovation, theoretical insight, and hardware-aware design. We hope this survey provides a solid foundation for future research into efficient, high-performance language modeling systems.

## Acknowledgments

This work was supported in part by 10.13039/501100001809National Natural Science Foundation of China under grant no. 62272264 and 10.13039/501100012166National Key Research and Development Program of China under grant no. 2020YFA0804503.

## Author contributions

Y.S. led the overall writing of the manuscript and was responsible for the section on linear attention, with assistance from Y.G. in developing specific subsections of the linear attention analysis. Z.L. and Y.Z. were responsible for the sparse attention section. B.D. and T.P. contributed to the section on pretrained LLMs with efficient attention. J.W. provided the original conceptualization, supervised the entire project, performed the final review, and acquired the funding.

## Declaration of interests

The authors declare no competing interests.

## Declaration of generative AI and AI-assisted technologies in the writing process

During the preparation of this work, the authors used ChatGPT, Gemini, and Deep Research in order to polish the language and improve readability. After using this tool or service, the authors reviewed and edited the content as needed and take full responsibility for the content of the publication.
